# Not Above the Law: A Legal and Ethical Analysis of Short-Term Experiences in Global Health

**DOI:** 10.5334/aogh.2451

**Published:** 2019-06-17

**Authors:** Virginia Rowthorn, Lawrence Loh, Jessica Evert, Eleanor Chung, Judith Lasker

**Affiliations:** 1 University of Maryland Baltimore, US; 2 University of Toronto Dalla Lana School of Public Health, CA; 3 Department of Family and Community Medicine at the University of California, San Francisco, US; 4 Maryland Court of Special Appeals, Clerkship Program, US; 5 Department of Sociology and Anthropology, Lehigh University, US

## Abstract

**Background:**

Persons from high-income countries have multiple opportunities today to participate in “short-term experiences in global health” (STEGHs) in low-resourced countries. STEGHs are organized through religious missions, service learning, medical internships, global health education, and international electives. An issue of increasing concern in STEGHs is “hands-on” participation in clinical procedures by volunteers and students with limited or no medical training. To address these concerns, best practices and ethical standards have been developed. However, not all STEGH organizations adhere to these guidelines, and some actively or tacitly allow unethical and potentially illegal practices.

**Objectives:**

This paper considers the legal framework within which STEGHs operate. It assesses whether certain STEGH practices break laws in the US and/or host countries or violate international “soft” legal norms. Two activities of particular concern are: practicing medicine without a license and drug importation and distribution.

**Conclusions:**

Many activities undertaken in STEGHs would be illegal if they took place on US soil. In addition, these same activities are often illegal in the host countries where STEGHs operate, although compliance is unevenly enforced. Many STEGH activities violate World Health Organization guidelines for ethical conduct in humanitarian activities.

**Recommendations:**

This paper encourages STEGH organizations to end unethical and potentially illegal activities; urges regulatory and non-regulatory stakeholders to alter policies that motivate participation in illegal or unethical STEGH activities; and encourages host countries to enforce their local and national health laws.

## Introduction

Unethical and potentially illegal clinical activities by untrained, undertrained, and unlicensed volunteers and students have become an unfortunate component of many “short-term experiences in global health” (STEGHs) undertaken in low-resourced countries by persons from high-income countries. Examples include:

- A STEGH organization’s website features a promotional video in which high school students describe delivering a baby, participating in an amputation, and helping to repair an ACL.^1^
- In a STEGH promotional video entitled “Lewis Does Brain Surgery,” a 19 year old student describes participating in spinal fusion and other surgical procedures, stating “now I feel like I can do a spinal fusion myself.”^2^
- An undergraduate student travels to Africa and, after observing 2 lumbar puncture procedures, is permitted by the physician in charge to perform more than 100 of these procedures on patients over a six-week time period.^3^
- A premedical student volunteering abroad does not speak or understand the native language of the physicians, other local health care workers, or patients, yet is permitted to diagnose and write a prescription for a patient. She “thinks” she has cleared the proper prescription dosage with the local physician; however, the dosage she writes is 100 times stronger than what should have been prescribed.^4^
- A group of undergraduates on an overseas learning experience is tasked with reading microscopic slides used to diagnose malaria. A couple of days later, someone realizes that they are reading the slides incorrectly and have misdiagnosed dozens of patients.^5^
- A 22-year-old US premedical student states during a volunteer experience in a Tanzanian hospital, “I’ll be damned if I leave Tanzania and haven’t delivered a baby.” Two days later, after a 15-minute lecture by a British midwifery student volunteer, he delivered a baby, unsupervised by Tanzanians.^6^

## Introduction

Overlapping 21st century cultural, economic, religious, and historical forces have combined to drive a greater participation in volunteering activities abroad for the purpose of service.^7^ Of note, the provision of health care by volunteers has become very popular with universities, independent study abroad and service learning operators, and non-governmental and religious organizations operating programs that send a massive outflow of high income country citizens to clinical settings in low- and middle-income countries (“LMICs”). These activities occur in various forms, including medical missions, volunteering, service learning, internships, global health education, international electives, and medical externships. In this paper, these activities are grouped under the umbrella term “short-term experiences in global health” (“STEGHs”).^8^ Participants range in training and background and include secondary school, undergraduate, and graduate students, as well as church group members and licensed professionals. Studies suggest each group possesses different primary reasons for participation.^9^

Regardless of the participants’ intentions, studies document numerous concerns with STEGH programs, including the strain that many place on health care personnel and patients in low-resourced medical settings; the lack of equity in bidirectional training opportunities; and the absence of ethical community engagement, capacity building, and sustainability practices evidenced by some programs.^10^ In particular, the ethical quandary presented by the “hands-on” clinical participation of volunteers, notably by students lacking medical training, as well as health care students and professionals practicing beyond the scope of their training, has come under significant scrutiny. Such participation creates a danger for patients, and equivalent opportunities would never be afforded to similarly unqualified individuals in the US.^11^ The tolerance for this approach to providing health care in LMICs stands in contrast to the outrage demonstrated when an 18-year-old in Florida was caught posing as a doctor and performing physical exams and offering medical advice. He was subsequently arrested and charged with a third-degree felony. The local sheriff stated via Twitter: “Just because you saw a season of Grey’s Anatomy doesn’t mean you could practice medicine.”^12^ Yet, as anthropologist Noelle Sullivan noted after the event, if the young man were “to fly to Tanzania, Cambodia, Bolivia, Honduras, Senegal, Nepal or any other so-called ‘developing country’, not only would he be able to practice medicine without a license…his actions would be celebrated.”^13^

Multiple sources are available that describe best practices and ethical standards to guide appropriate conduct in health-related settings abroad.^14^ The core guiding principle of these various frameworks is the primacy of host community needs and interests over those of STEGH participants. However, not every STEGH organization is aware of, or adheres to, these guidelines, and some organizations actively or tacitly allow unethical and potentially illegal practices. Certain US-based organizations brazenly market STEGH programs in LMICs as a means to circumvent regulations at home, with one stating in its online appeal directed to high school students that “… overseas it’s a completely different situation, and in many countries these [hands-on learning] experiences can be arranged in ways they never could in the US.”^15^ Taken in sum, these concerns raise such serious questions about the ethics of STEGH programs that many have questioned whether some of these organizations and their participants are breaking laws in the US or in the host countries where their activities occur.

This paper considers the legal framework in which STEGHs operate, and the risks and concerns associated with a disregard for the laws of home and host country. As this paper will demonstrate, many activities undertaken by STEGHs would be illegal if they took place on US soil. Moreover, these same activities are usually illegal in the host countries where STEGHs operate, although compliance with local laws is often not enforced. The authors argue that STEGH organizations should adhere to, and be held accountable for, compliance with all applicable laws. Further, we argue that STEGH organizations are obligated to conform to the normative standards set forth by the World Health Organization (WHO), the US Food and Drug Administration (FDA), and prominent non-governmental entities engaged in global health.

## Background on STEGH Activities and Supervision

The health care-related activities undertaken by volunteer participants of STEGH organizations vary significantly in nature, invasiveness, and supervision. They range from shadowing health care providers, providing patient education or community health workshops, working with human or animal cadavers, observing surgeries, distributing over-the-counter and prescription drugs and medical supplies, and engaging in hands-on clinical patient care.^16^ Patient care activities include observation, obtaining patient histories, assessment, physical exam, non-invasive physical maneuvers, diagnosis, counseling and guidance, issuing prescriptions, procedural treatments (e.g., biopsy, suture, needles, foreign body removals, wound care), intimate examinations, surgeries and obstetric deliveries.^17^ The manner and degree of medical supervision overseeing these activities can vary, with some organizations sending licensed physicians or other health professionals as part of the team, other organizations working with local health care providers, and still other programs sending groups comprised wholly of students or other untrained volunteers.^18^ Of note, even when licensed US or local health care providers are involved, legal and ethical boundaries may be crossed when providers work outside their scope of practice or are unfamiliar with the specific context (geographic, cultural, resource-level) where the patient care is occurring.^19^

At times, in order to meet volunteer desires, some STEGH organizations are known to explicitly or tacitly allow untrained volunteers to engage in activities across the full spectrum of patient care.^20^ Host country medical needs coupled with scarce resources, norms of hospitality, unequal power dynamics, and financial agreements with STEGH organizations may further encourage untrained volunteers to engage in such care.^21^ Many US participants specifically want hands-on clinical experience and are willing to pay sending organizations for that opportunity. The financial incentive that encourages the creation of overseas clinical opportunities for students cannot be ignored, with STEGH organizations charging participants up to $6,250 for a 16-day program.^22^

STEGH organizations often take over-the-counter and prescription drugs to donate or distribute, in their original packages or informally repackaged in resealable bags.^23^ These drugs are either purchased in bulk expressly for the STEGH project by someone with prescribing authority, or they are donated by pharmaceutical companies, distributers, or clinics.^24^ Donated drugs sometimes include expired drugs or drug samples.^25^ In some cases, the collected or purchased drugs are given to unlicensed volunteers to take to the host country site.^26^ Finally, there are some organizations in the US that provide pre-packaged drug kits that organizations can purchase online for the explicit purpose of administering them in LMICs.^27^ On site, drugs are often distributed to patients by volunteers who may not have medical training, often through a language barrier, a limited understanding of the patient’s history and other critical context, and with limited or no follow up.^28^

Although some organizations work ethically with local health care systems and communities to provide needed medical services, many others comprise an industry built around providing STEGH opportunities while disregarding or ignoring LMIC health care systems, populations, and laws.^29^ Taken in sum, the various costs and burdens of poorly-thought-out STEGHs to host communities is considerable.

## Legal Frameworks for STEGH Activities

Laws relating to health care delivery reflect societal notions of how patients, health professionals, and health care organizations should be treated, and reflect ethical, safety, and economic considerations. The US has strict laws relating to, among other health-related matters, professional licensure, standards of care, and the distribution and use of pharmaceuticals. These laws make it virtually impossible – as one would hope and expect – for untrained individuals to engage in patient care and, in some cases, even observe clinical care without training in patient privacy laws and other subjects.^30^ These laws are why one STEGH organization states on its website,

Experiencing “The Big 3” [working on human cadavers, observing surgery in the operating room, and shadowing physicians] in the United States used to be difficult but now it’s almost impossible. Why? Because of our privacy laws, insurance rules and lawyers at hospitals and medical schools that don’t want to risk a lawsuit.^31^

Many in the global health field find it objectionable that some US-based STEGH organizations facilitate activities overseas that would be illegal in the US and look to US law as a way to stop them. However, with narrow exceptions, the Supreme Court has repeatedly held that US law does not apply to activities that take place outside of US territory except in very narrow situations defined by Congress.^32^ Additionally, in the US, matters of occupational regulation, such as licensure and scope of practice, are governed by the laws of the 50 states, which generally apply only within a state’s borders. Accordingly, the primary legal framework to which STEGH organizations must adhere is the law of the host country, and enforcement thus falls on the shoulders of the host country’s authorities. However, many STEGHs operate in a legal gray area in host countries – neither inquiring about local laws nor having the laws enforced against them – a situation created and perpetuated by poor communication, willful ignorance, lack of patience with unfamiliar bureaucratic systems, assumptions about what the law is or should be, and power imbalances.^33^

Laws articulated by governments are only one way in which individual and organizational obligations arise. Where formal or “hard” law does not reach, norms articulated by representative international bodies can guide action. Often called “soft” law because it is non-binding, international guidelines indicate the objectives and principles of the international community.^34^ WHO is the global leader in setting standards, underpinned by science, ethics, and human rights, expected by the world community when engaging in health-related activities. WHO does not have an enforcement arm, but rather depends on international consensus, partnership, education, and pressure to ensure compliance with its guidelines. Thus, while these guidelines do not have the force of law, they are the written embodiment of international standards developed through a consensus-building process.^35^ Hence, STEGH organizations that do not follow the guidelines are essentially acting in a rogue manner vis-à-vis the community of nations.

## Legal Concerns Raised by STEGH Missions

The following section of the paper provides a detailed description of several activities undertaken by STEGH participants that are discouraged by WHO and other influential organizations; not allowed in the US; and, based on an analysis of laws in several countries that host STEGH programs, violate the law in host countries. The authors do not tackle all the areas in which STEGH organizations may be violating host country laws, such as those relating to immigration and visa regulations, research ethics, and patient privacy.^36^ Adherence to these rules should also be prioritized by STEGH organizations.

### Licensure Laws

To protect patients and ensure that health care providers meet the standards of their profession, state licensure is required to practice any of the regulated health professions in the US. Focusing on medicine (although the law is similar for other health professions), the “practice of medicine” is defined by individual state law, but generally means “to engage, with or *without compensation*, in medical diagnosis, healing, treatment, or surgery.” (emphasis added)^37^ Physicians must obtain state licensure directly from the appropriate medical board(s) in all the states in which they treat patients. As to medical trainees (residents), about half the states require residents to obtain a state trainee license, while the other states allow medical schools or affiliated hospitals to submit the names of all residents to the state medical board.^38^

Medical boards also have authority over medical students’ clinical practice, but do not require them to obtain state medical licenses, provided students interact clinically with patients within the confines of their programs of study, supervised by faculty, and subject to medical board regulation and oversight.^39^ Individuals with no medical training, including high school and undergraduate students, are never allowed to practice medicine in the US.

Similarly, in countries around the world, a medical license bestowed by a governmental entity or professional association is required to practice medicine and is granted upon evidence of the entity’s determination of sufficient medical education and training. Although not exhaustive, the authors’ research concluded that virtually all countries have a medical licensure framework. The global organizing body of medical boards, International Association of Medical Regulatory Authorities (IAMRA), has 112 member boards from 47 countries, many of which are frequent medical mission destinations, including Ghana, Kenya, and Tanzania.^40^

Around the world, most medical boards, including boards in the US, provide temporary licensure to accommodate visiting physicians on short term missions. For example, the temporary medical licensure law in Kenya specifies that

Any practitioner not registered in the Republic but who, having valid qualifications from a different country, and who is desirous of giving medical or dental services in the course of any humanitarian or other valid cause, shall be required to obtain a licence upon payment of the prescribed fees, which licence shall be valid for such period as shall be determined by the Board, subject to a maximum period of twelve (12) months and subject to a renewal upon expiry.^41^

As another example, in 2018, the ten ASEAN (Association of Southeast Asian Nations) member countries affirmed the importance of temporary licensing for physicians on humanitarian missions and the organization’s website sets forth the different country requirements to obtain such licensure.^42^ In Malawi, visiting physicians can obtain temporary registration (licensure) for one year, but may be required to attend an approved hospital orientation if the applicant does not demonstrate sufficient training in local disease conditions. The Malawian Medical Council’s website specifically addresses why registration is required by foreign physicians:

Some people have questioned the need to register with the Medical Council of Malawi. The answer is as follows: Registration is a legal requirement according to the Medical Practitioners and Dentists Act (Cap 36:01, Laws of Malawi). It is illegal and punishable by law for one to engage in medical practice without registering with Medical Council of Malawi. The law requires every medical practitioner to register with Medical Council as an indication that the Council vouches that practitioners have the required qualifications and experience for their calling.^43^

Overseas health professional schools and clinical sites usually have strict rules governing trainees’ and students’ clinical interaction with patients and supervision requirements. For this reason, it is important to inquire about applicable rules, to follow those rules, and to consider affiliation agreements with partner universities and hospitals to support mutual understanding, agreement, and compliance between the STEGH organization and the partner.

Importantly, an environmental scan conducted by the authors of international medical boards, although not exhaustive, did not find a single medical board that endorses, or provides a licensure category for, individuals lacking a medical license in another jurisdiction or who are not enrolled in a formal medical course of study.

In the US, practicing medicine without a license is a legal violation that is punishable by fines or criminal sanctions.^44^ Likewise, the unlicensed practice of medicine is an offense that carries strong penalties in most countries for both the individual and employer. For example, Nigerian law specifies:

Any employer who engages the services of an unlicensed Doctor is liable in law for criminal breach of the law and may be prosecuted. To this end, it is the responsibility of every employer of Medical Practitioner or Dental Surgeons to ascertain and ensure that the persons they employ are registered and licensed by the Council, whether they are Nigerians or Non-Nigerians, and in whatever place of employment within the territory of the Federal Republic of Nigeria.^45^

Many STEGH organizations and participants do not inquire about, nor seek, temporary licensure or appropriate training affiliation agreements in host countries. Licensed US health care providers often assume, or are told by the US-sponsoring organization, that their US licensure is sufficient. When inquiries are made, many are told that the sponsoring organization or local clinical site is “taking care of it” or that it is too administratively burdensome to get a license, and that engaging in that process will take medical care away from those who need it. To the extent that an individual practices medicine without a license from, or the specific authorization of, the host country’s licensing body, the individual is likely violating the law of the host country, even if the unlicensed activities appear to be tacitly endorsed in a particular clinical setting. It is therefore the legal and ethical obligation of anyone who conducts clinical activities abroad to make their own inquiries regarding licensure or medical education training standards to host country authorities, even in the emergency relief context (with common sense exceptions in critical situations). This default position is vital for patient safety and health system planning purposes.

In addition to demonstrating respect for local laws and communities, adherence to host country licensure laws ensures that STEGH participants are not placed in situations where they are asked to take on more than they are trained to do, and more than they want to do. Researchers have documented the potentially devastating emotional consequences of giving increased medical responsibility to trainees in situations where errors are least likely to be noticed or remediable.^46^ Additionally, placing students in this situation can have legal ramifications for STEGH organizations, as a United Kingdom case demonstrates. A British student sued Frontier, the non-profit organization that arranged for her post-A-level volunteer medical trip abroad.^47^ When the student complained to Frontier that she was ill-equipped to help in the busy host country hospital, Frontier terminated her contract, leaving her stranded in Madagascar. The student and Frontier settled out of court.

On occasion, in order to respect and/or circumvent local licensure laws, some STEGH organizations offer volunteers the opportunity to observe or shadow health care providers in LMIC settings. This practice is not without concerns, however, as observation and shadowing without training or patient consent is intrusive and inappropriate for students who offer nothing in return for the burden they place on stressed health care systems and breached patient privacy.^48^ High school and college students are prohibited from clinical observation in certain jurisdictions in North America, including in British Columbia.^49^ A recently published code of conduct for observing physicians in a clinical setting noted significant concerns and suggested that those interested in shadowing physicians first complete HIPAA training, sign a confidentiality agreement, and complete infection control requirements.^50^ The concerns that led North American physicians to suggest a code of conduct for observation are equally – if not more – present in LMIC settings and should be factored into STEGH program development.

Although anecdotal information indicates that obtaining host country medical licensure is rare among STEGH participants, the importance of adhering to these laws and regulations should not come as news to STEGH organizations. Prominent organizations and advocates in the global health space have proclaimed the need to do so in multiple fora. Some examples include the 2010 Working Group on Ethics Guidelines for Global Health Training (WEIGHT) which urged sponsored programs to “comply with licensing standards.”^51^ The American College of Physicians position paper on STEGHs noted that “[l]icensing requirements must be adhered to.”^52^ The Association of American Medical Colleges (AAMC) published guidance noting that “[e]ven if a local health care provider is supervising your interactions with patients or says that it is acceptable for you to perform a procedure, violation of local laws may still be a punishable offense.”^53^ A consortium of Christian medical mission leaders in a document describing best practices for missions stated that “care provided by health missions must meet the legal requirements and medical standards and practice guidelines of the host country.”^54^ Thus there is little merit to the argument that this foundational pillar of responsible engagement is a novel concept embraced only by a minority of STEGH organizations.

### Drug-Related Legal Concerns

The legal implications of STEGH drug practices are unclear for several reasons. First, the stockpiling and distribution practices of STEGH organizations involve the intersection of multiple US regulatory frameworks, including those that govern physician self-prescribing, drug donations, and drug exportation. Secondly, although some portion of the drug-related activity of STEGHs takes place in the US (e.g. purchase, repackaging), the drugs are then taken outside of US borders where US law no longer applies, as the drug and pharmacy laws of the host country prevail. Many STEGH organizations violate US export and repackaging laws, and subsequently violate the prescribing and distribution laws of the host country, but enforcement is not triggered in either setting. This is due to a lack of enforcement funds, in both the US and abroad, as well as drug import and export legal paradigms that are focused on the pharmaceutical industry rather than volunteerism.

Stockpiling drugs to take overseas for STEGH missions takes place in different ways. In some cases, US physicians use their prescribing authority to purchase drugs for overseas projects.^55^ State practice laws generally prohibit the prescription of medications to a person other than the one to whom the medications will be administered (a process referred to as third-party prescribing) or to a person with whom the prescriber does not have a prescriber-patient relationship.^56^ This became a topic of national discussion recently when several municipalities sought to give the prescription anti-opioid Naloxone to close associates of opioid users in anticipation of an overdose.^57^ In that situation, the prescribing physician would have no relationship or knowledge of the individual who would ultimately be given the drug, a corollary situation to prescribing/purchasing drugs for unknown individuals overseas.

If the drug-related practices of STEGH organizations, including writing prescriptions and purchasing drugs for unknown patients, occurred in the US, those involved would be at risk for prosecution or discipline by the state medical board. However, when the drugs are taken overseas, state medical boards have no direct authority to discipline their licensees. Organizations that market drugs for medical missions require purchasers to certify that the drugs will be used exclusively outside the US, likely to avoid liability for breaking US state prescribing and distribution laws.^58^

Some STEGH organizations rely on donated, rather than purchased, drugs. State law strictly regulates donation programs for unused prescription drugs that will be re-dispensed to patients.^59^ These laws were passed to support donation while protecting patients from adulterated, expired, or other inappropriate donations. As of mid-2016, 42 states had laws establishing drug redistribution programs.^60^ Many state programs specifically allow donations to charitable organizations under certain common parameters:

- No controlled substances are accepted.
- No adulterated or misbranded medications are accepted.
- All pharmaceuticals must be checked by a pharmacist prior to being dispensed.
- No expired pharmaceuticals are allowed (they often must have six-months or more before expiration when donated).
- All pharmaceuticals must be unopened and in original, sealed, tamper-evident packaging.^61^

Drugs donated to STEGH programs and used overseas are not covered by state donation programs and therefore are not subject to the protections offered by these programs. This is not to say that all drugs used by STEGH programs do not meet these standards; rather, states cannot enforce these protective standards overseas and the discretion to meet the standards therefore rests with the STEGH organization or host country authorities.

The use of donated drugs for humanitarian purposes is specifically discouraged by WHO and FDA. In 2010, WHO published “Guidelines for Medicine Donations” that were written in coordination with the world’s largest international health organizations, including several UN agencies and the World Bank.^62^ The document specifically covers drug distribution by private volunteer organizations and other groups that could be considered STEGH organizations, and provides examples of poor medicine donation practices. The guidelines unequivocally state four core principles that form the basis of good medicine donation practices, several of which are unheeded by many STEGH organizations:

- Donations of medicines should benefit the recipient to the maximum extent possible. All donations should be based on an expressed need. Unsolicited medicine donations are to be discouraged.
- Donations should be given with due respect for the wishes and authority of the recipient, and in conformity with the *government policies and administrative arrangements* of the recipient country. (emphasis added)
- There should be effective coordination and collaboration between the donor and the recipient, with all donations made according to a plan formulated by both parties.
- There should be no double standard in quality. If the quality of an item is unacceptable in the donor country, it is also unacceptable as a donation.^63^

WHO specifically discourages donations of returned and expired drugs and drug samples stating, “[d]onating returned medicines (unused medicines returned to a pharmacy for safe disposal, or free samples given to health professionals) is an example of a double standard because in most countries their use would not be permitted owing to regulations on quality control.”^64^

In a guidance document that references the WHO guidelines, FDA urges individuals and small groups to refrain from exporting donated prescription drugs (including expired drugs and samples) to other countries, recommending that “only drug manufacturers donate drug products so they can assure a drug’s quality, safety, and efficacy, including the proper storage and transportation of the drug.”^65^ The FDA guidance further states that organizations sending their own medical supplies should ensure that “all products contained in the cache are in compliance with the Federal Food, Drug, and Cosmetic Act (FFDCA) and/or Public Health Service Act.”^66^ Notwithstanding this last admonition, it remains unclear if, and how, FDA would enforce such standards once the drugs are taken outside the country.

In the case of controlled substances, US enforcement is more robust. The US Drug Enforcement Agency (DEA) has an expedited waiver process for “humanitarian aid situations.”^67^ Exporting controlled substances without such a waiver can result in federal prosecution. In 2010, a US dentist was arrested at the Nashville, Tennessee airport and ultimately fined for attempting to take diazepam, hydrocodone, and oxycodone to Haiti on a mission trip.^68^

A separate drug exportation rule enforced by US Customs and Border Protection (USCBP) applies to drugs taken out of the country for “personal use” which does not squarely fit with STEGH drug distribution activities. The USCBP advises travelers on its website that they should “travel with no more than personal use quantities, a rule of thumb is no more than a 90 day supply” and that “prescription medications should be in their original containers with the doctor’s prescription printed on the container.”^69^ There is no publicly available guidance regarding the actions the agency will take for violating this “rule of thumb” nor cases the authors could identify regarding actions against violators.

On the host country side, every country has different rules regarding importation of prescription drugs. At a minimum, most countries require a packing list that describes what is being imported, and, in the case of donations, paperwork confirming the drugs will not be sold commercially.^70^ Many countries prohibit importation of expired drugs.^71^ WHO guidelines regarding donated medicine set forth numerous examples of how medicine donations have violated recipient country law, including medicines that did not comply with locally agreed policies and standard treatment guidelines; donated medicines using trade names that were not registered for use in the recipient country and without an International Nonproprietary Name (INN) or generic name on the label; and medicines donated without the required host country documentation.^72^

WHO specifically urges donors to “respect the laws, regulations and administrative procedures of the recipient country in all instances.”^73^ The WHO guidance further states that donors should consult the relevant ministry in the recipient country for special documentation requirements in order to ensure smooth reception and clearance of the drugs and, where appropriate and whenever possible, donated medicines should have clearly labelled (in the appropriate local language) indications and counter-indications for pregnant and lactating women, for children, and for persons suffering from other health conditions. This extensive WHO guidance leaves little question that STEGH organizations that deliver drugs to host countries without going through required host country procedures are not only breaking local laws, but acting outside international norms of good practice.

### Negligence and Medical Malpractice in Host Countries

Another legal realm implicated by the work of STEGH organizations is medical malpractice. Given the widespread observation that many STEGH organization participants provide clinical care beyond their level of training, with varying degrees of supervision, and limited local language ability, medical accidents are likely taking place in LMIC settings whether or not cases are reported or litigated.^74^ A recent article documented examples of medical negligence by US STEGH participants in LMIC settings.^75^

In the US, medical malpractice takes place when a health care provider acts negligently (outside the standard of care) in rendering care and that negligence results in injury and damages.^76^ Although widely viewed as overused in the US, the ability to sue a physician for negligent care is one avenue to ensure that health care meets a threshold level of quality. In the STEGH setting, the visiting provider is most likely unfamiliar with the standard of care in the host country, while similarly, host country providers may be unfamiliar with medications and treatments provided by volunteer health care workers, creating a situation ripe for medical negligence to occur.^77^

Overall, medical malpractice laws are weak in lower resourced countries and patients often have problems with access to justice for malpractice claims.^78^ For this reason, parties in developing countries often prefer to settle their disputes informally. There is limited research regarding judicial actions taken against STEGH participants for medical negligence. A 2010 article in the American Academy of Pediatric News stated that “extensive searches of U.S. and international case law have yielded no active or past cases of medical malpractice against a physician who has volunteered internationally,”^79^ but a study by a religious mission organization of its members found a handful of malpractice cases and settlements.^80^ However, the World Medical Association warned in 2015 that “[a] culture of litigation is growing around the world … [s]ome National Medical Associations report a medical liability crisis whereby the lawsuit culture is increasing.”^81^ This should serve as a warning to individuals working beyond their scope of practice or training in LMICs and the organizations that support them.

In one of the few articles looking at the liability risk for US physicians overseas, the author concluded that many countries have laws that protect physicians who act in good faith as volunteers or during emergencies.^82^ However, the author noted that if such “good faith” laws exist, they usually apply only *if the provider is “properly licensed and certified to perform the task required*.” (emphasis added)^83^ According to one source, most physicians participate in medical missions without liability coverage because most traditional policies do not cover international volunteer work, and coverage through specialized policies is expensive and often limited.^84^ Many sponsoring organizations do not provide medical malpractice coverage, and some go so far as to state that coverage is not necessary because the likelihood of a lawsuit is negligible.^85^ Practicing physicians sometimes extend their current professional liability coverage or purchase short-term coverage for overseas STEGH programs. Whether extending or purchasing coverage for an overseas project, the exclusion clauses of some policies may invalidate coverage if the STEGH participant is not *properly licensed and authorized to provide the types of health care services offered*. (emphasis added)^86^ As such, these professional liability insurance considerations support the importance of obtaining host country licensure.

## Recommendations

Moving forward to a time when all STEGH organizations and their participants decline to participate in unethical and illegal activities will take education, persuasion, discussion, and consensus building, but it is achievable. Almost all STEGH organizations and participants are motivated by the laudable goal of sharing their time and energy with others for good. Global health stakeholders need to facilitate conversations about how to harness that energy in support of global health equity over the long haul, rather than spend millions of dollars on medical missions that give paying participants a meaningful experience without supporting sustainable health care system strengthening. To that end, the authors offer the following recommendations.

### STEGH organizations must make a commitment to follow host country laws

The path to global health equity is complex and time-consuming and should be driven by the articulated needs of vulnerable communities balanced with legal, ethical, and safety considerations. The authors acknowledge that many vulnerable communities actively welcome the free medical care and drugs provided by STEGH organizations. For communities that are neglected by their own governments and legal systems, circumventing local regulations may seem like the only way to meet the expressed needs of communities. However, a baseline foundational pillar of all STEGH organizations should be knowledge of, and adherence to, host country laws. We assert that adherence to US and host country law, as well as international ethical standards, is eminently achievable, and that indeed some STEGH organizations already do so. Understanding and abiding by the laws of foreign countries is the legal, ethical, and moral obligation of any organization introducing US citizens to non-US clinical settings. It is incumbent upon visiting organizations to work with national authorities to determine which local laws are impacted by STEGH programs, including laws relating to licensure, graduate medical education, clinical supervision, prescribing, and over-the-counter drug distribution. If it emerges that a STEGH organization does not have the time, resources, or ability to follow local laws, the proposed program should be substantially altered or terminated.

In settings where it is demonstrably impossible or unreasonable to follow local health laws, STEGH organizations should work with other STEGH organizations, hospitals, health science schools, or community organizations in that locale to form a consortium for the purpose of developing appropriate practice and supervision standards for STEGHs operating in that region. Such an effort would ensure that programs meet agreed upon standards, rather than arbitrarily decide whether or how to follow applicable law.

### Host country authorities should be encouraged to enforce local laws against STEGH organizations when appropriate

US citizens and organizations involved in clinical care overseas should engage with local authorities and support their enforcement efforts by including such officials in the co-creation of health-related initiatives, even if doing so delays, alters, or prohibits a planned program. The best way to engage local officials is through hospital administrators, health science university programs, and/or the Ministry of Health, as these entities are best suited to help visitors understand local health laws and identify regulators. This recommendation presupposes a scenario in which host country authorities are involved in some way in the provision of health care to a particular population, which may not always be the case. STEGH organizations must work thoughtfully with community leaders to determine what regulatory authorities are engaged in local health care and what laws apply to visiting health care organizations. It is never okay to make assumptions or conclusions about host country laws without a diligent investigation that involves individuals who speak and read the applicable language(s) and are familiar with the applicable laws.

### Universities must limit the opportunities and incentives for STEGH participants to engage in illegal activity overseas

There are a number of factors motivating US citizens to engage in medical work overseas, one of which is the hope that the experience will result in admittance to university programs or enhance employment prospects. University leaders and advisors must educate their students about ethical engagement with communities, and encourage students to work with organizations that adhere to high ethical standards. Some STEGH promotional materials explicitly state that overseas health care experience will enhance a student’s application materials. Admissions departments should not inadvertently celebrate unsafe, unethical, or illegal activities during the application process.^87^

As an example of good practice, the University of Minnesota is addressing these issues in a variety of ways. Undergraduate students applying to register as a student organization in the Student Unions and Activities Office must sign a document affirming that organization officers and members will not participate (in the US or overseas) in health delivery activities. Students are provided with an extensive list of activities to guide their response on behalf of the registering organization.^88^

Registered student organizations are explicitly considered “independent and autonomous from the University and are responsible for managing their own affairs” and are not covered by the University’s General Liability Insurance^89^ but, if they want to use university spaces for meeting and events, organizations must be registered. Students in health professional programs must join an entirely different type of student organization (a Campus Life Program or CLP) that is housed formally in a health profession program or school. CLPs are required to comply with all university policies (including policies relating to overseas activities which prohibit inappropriate patient activities) and are subject to oversight. In this creative way, the university can enforce ethical and legal practice by both undergraduate and graduate students.

The university also created the Global Ambassadors for Patient Safety (GAPS)^90^ online workshop for students who are going abroad to volunteer in a health care setting. The program, now available via open access online, includes modules that discuss what students can and cannot do abroad and why. It also gives students a readily available explanation and excuse not to engage in clinical care if asked to do so.

### US state medical licensure boards should support efforts to end unlicensed practice and illegal activity in other countries

US medical licensure boards can serve as allies to stop STEGH organizations from violating medical practice and drug distribution laws of host countries and play a role in educating current and future licensees regarding the lack of professionalism and risk to patients demonstrated by such practices. Many individuals who participate in STEGHs are either current or future licensees. Medical boards are uniquely qualified to speak to the importance of licensure in all settings, as well as the link between licensure, patient safety, and professionalism. Boards frequently educate their licensees on topics of importance through newsletters, webinars, and training sessions, and should be encouraged to share the recommendations set forth in this paper with their licensees.

If licensure boards wanted to take a more aggressive stance on the issue, they could ask applicants (both first-time and those seeking renewal) to document clinical service or educational experiences overseas, including dates of the activity, actions undertaken during the activity, and the name of the sponsoring organization. Boards have broad authority to determine “fitness to practice” when granting licenses, which enables inquiries into prior educational and clinical experiences.^91^ Such a question, depending on the applicant’s answer, could trigger a request for documentation, such as evidence (or lack thereof) of clinical activity undertaken without an appropriate license or university affiliation agreement. Boards may not have the resources to effectively investigate violations (i.e. interviewing out of country witnesses/patients, getting out of country witnesses to testify at a hearing, and obtaining out of country medical records) but could determine that specific responses to this question will require applicants to take an in-person or online course in ethics.

The US-based Federation of State Medical Boards and the International Association of Medical Regulatory Authorities should also consider taking a position on this issue by educating their member boards and developing strategies to help end the practice of unlicensed care by STEGH participants by, for instance, adopting model licensure application questions and responses to help boards address this issue.

### FDA should publish guidance specifically addressing STEGH drug-related practices

FDA has been an ally in the effort to ensure that US organizations follow best practices for drug procurement and distribution in international settings via its guidance “Questions and Answers for the Public: Donating Drugs to International Humanitarian Relief Efforts.”^92^ Although helpful, the guidance is geared to large scale relief efforts such as natural disaster response efforts. It would be extremely helpful for FDA to engage in a consultation process with global health advocates in the US and overseas to create a guidance document specifically directed to STEGH organizations. Such a guidance document should set forth best practices, including the recommendation that all host country drug importation and distribution laws be followed.

### A consortium of universities or global health advocacy groups should create a certification program and/or seal of approval for STEGH organizations

A potential avenue to address under-regulation of STEGH organizations is a seal of approval or certification program to set standards for the industry and help consumers choose an organization that adheres to high ethical standards. Certification programs, which typically allow complying businesses to post the certifying organization’s seal, are relatively common in the consumer market, primarily used by trade organizations and coalitions to indicate that a product or service has met a certain level of safety, quality and/or efficacy, usually in compliance with written standards. Relevant certification programs already in existence are those for education abroad programs,^93^ summer camps,^94^ and hospitals.^95^ A consortium of universities or global health advocacy organizations could launch a certification program in which STEGH organizations can self-report compliance with standards or (moving up the continuum of complexity and cost) allow the organization to be reviewed by the certifying organization. A fee is often charged to an entity desiring certification to support the administrative costs of the certifier, but it is essential that the certifying agency be independent and not issue certificates simply for payment of a fee. A certifying organization might also consider blacklisting certain organizations for unethical conduct although care would have to be taken to document concrete evidence of bad practices to avoid litigation instigated by blacklisted organizations.

**Recommendation Summary**

- STEGH organizations must make a commitment to follow host country laws
- Host country authorities should be encouraged to enforce local laws against STEGH organizations when appropriate
- Universities must limit the opportunities and incentives for STEGH participants to engage in illegal activity overseas
- US health licensure boards should support efforts to end unlicensed practice and illegal activity in other countries
- FDA should develop a guidance specifically addressing STEGH drug-related practices
- A consortium of universities or global health advocacy groups should create a certification program and/or seal of approval for STEGH organizations

## Conclusion

Interviews with health professionals in host countries reveal a growing concern with competition from foreign volunteers who can provide free services and who often disregard or disrespect existing local expertise.^96^ As the medical workforce in LMICs grows in numbers, experience, and organization, and as local pharmaceutical industries grow, the likelihood is great that host countries will become less likely to excuse or ignore visiting missions that do not gain legal approval for their activities. Anecdotally, many LMIC medical boards and Ministries of Health are developing ways to stop these practices. Thus, in addition to the legal and ethical reasons for compliance cited in this article, organizations planning STEGHs should be prepared for this coming effort underway in recipient countries.

Unethical and illegal activities by some STEGH organizations may exist because of an outdated charity model of aid that ignores the complex long-term needs of LMIC health care systems, populations, and laws. Some organizations may also believe that bypassing burdensome legal constraints is a necessary short-cut to meet the needs of underserved communities. We argue that this view is short-sighted and not best in the long run for either volunteers or hosts. The opportunity costs of creating clinical opportunities and experiences with US participants at the center instead of more sustainable, outcomes-oriented community-based initiatives is considerable and troubling. The recommendations set forth in this paper are designed to move US volunteers and organizations to embrace a model of training and service that puts the needs of communities and health care systems and respect for their laws at the fore – a model that has the best chance at achieving sustainable health care equity across the globe.

## Footnotes

Homepage, Future Docs Abroad, https://allaccessmed.com/, archived at https://perma.cc/F4UT-YNY5 (last visited March 22, 2019).

Episode 2: Lewis Does Brain Surgery, Future Docs Abroad, https://allaccessmed.com/episode-2/ archived at https://perma.cc/44UQ-Y45M (last visited March 22, 2019).

Evert J, et al., Do You GASP? How pre-health students delivering babies in Africa is quickly becoming consequentially unacceptable. Health Advisor 2015; 61(35): 61–65.

Ibid.

Ibid.

Sullivan N. Posing as a doctor is illegal unless you go to the “developing world.” Campus Compact online blog July 22, 2016, 016, https://compact.org/posing-as-a-doctor-is-illegalunless-you-go-to-the-developing-world/.

Loh L, et al. Short-term global health experiences and local partnership models: A framework, Globalization and Health. 2015; 1(11): 1–7, https://globalizationandhealth.biomedcentral.com/track/pdf/10.1186/s12992-015-0135-7.

Melby MK, et al. Beyond medical “missions” to impact-driven short-term experiences in global health (STEGHs): Ethical principles to optimize community benefit and learner experience. Academic Medicine 2016; 633(91): 633–38.

See Evert, supra note 3; Career opportunities in global health: A snapshot of the current employment landscape. 5 J. Global Health. 2015; 1: 1–5; Evans T, et al. Using Maslow’s Hierarchy to highlight power imbalances between visiting health professional student volunteers and the host community: An applied qualitative study. Education for Health. 133(30): 133–39 (Stating that motivations include gaining experience to bolster higher education applications and employment prospects; meeting graduate school experiential learning requirements; or a desire to help, serve, educate, build capacity, or share the gospel in less-resourced settings).

See, e.g., Lasker J, et al. Guidelines for responsible short-term global health activities: Developing common principles. Globalization and Health. 2018; 1(14): 1–9. Trop Dis Travel Med Vaccines. 2017; Bauer I. More harm than good? The questionable ethics of medical volunteering and international student placements, Trop Dis Travel Med Vaccines. Published online 2017 Mar 6.

Hudspeth J, et al. Reconfiguring a one-way street: A position paper on why and how to improve equity in global physician training, Academic Medicine. November 2018.

See Sullivan, supra note 5 and see Michelle Quesada, “Fla. teen doctor released from jail,” Feb 16, 2016, available at https://www.ktnv.com/now-trending/malachi-love-robinson-male-arrested-for-practicing-medicine-without-a-license.

See Sullivan, supra note 6.

See, e.g., Guidelines for Premedical and Medical Students Providing Patient Care during Clinical Experiences Abroad, Association of American Medical Colleges (2011) https://www.aamc.org/download/181690/data/guidelinesforstudentsprovidingpatientcare.pdf; Dainton C, et al. A protocol to develop practice guidelines for primary care medical service trips. Annals of Global Health. 2016; 678(82): 678–685; Loh, supra note 7; Melby, supra note 8; Lasker, supra note 10.

The Future Docs Abroad Experience, Future Docs Abroad, https://allaccessmed.com/about/. Accessed Nov. 11, 2018.

Bradke A. The ethics of medical brigades in Honduras: Who are we helping? 2009 (unpublished master’s thesis, University of Pittsburgh) (http://d-scholarship.pitt.edu/8620/).

Ibid.

Ibid; Lasker J. Hoping to help: The promises and pitfalls of global health volunteering (2016); Sullivan N. International clinical volunteering in Tanzania: A postcolonial analysis of global health business. Global Public Health. 2018; 310(13): 310–324; Bauer I. More harm than good? The questionable ethics of medical volunteering and international student placements. Tropical Disease Medicine and Vaccines. 2017; 1(3): 1–12; Sykes KJ. Short-term medical service trips: A systematic review of the evidence. American J. Public Health. 2014; 38(104): 38–48; Martiniuk ALC. et al. Brain gains: A literature review of medical missions to low- and middle-income countries. BMC Health Service Research. 2012; 678(12): 678–685.

Doobay-Persaud A, Evert J, et. al. A mixed methods study of health professionals’ perceptions of and experiences with performing outside scope of training (POST) during short-term experiences in global health (STEGHs): The Predicament of POST (submitted, 2019).

See Bradke A, supra note 16; see Sullivan N, supra note 6.

Smaldino A, et al. Clear as mud: Voluntourism, power dynamics, and global health. In Global Health Experiential Education: From Theory to Practice (N. Arya and J. Evert, eds., 2017).

FREQUENTLY ASKED QUESTIONS: BELGRADE, SERBIA, FUTURE DOCS ABROAD 3 2008, http://allaccessmed.com/wp-content/uploads/2017/09/Serbia-FAQ-1.pdf, archived at https://perma.cc/CMS9-ZQMD.

See Lasker, supra note 18; Bridge to Health, Facebook (Jan. 21, 2018), https://www.facebook.com/BridgeToHealthOrg/photos/a.442915812422463/1513443562036344/?type=3&theater; Bradke A, supra note 16.

See Lasker, supra note 18.

World Health Organization. Guidelines for Medicine Donations, World Health Org. 2011, http://apps.who.int/iris/bitstream/handle/10665/44647/9789241501989_eng.pdf;jsessionid=23F7DA3C2136B6D735AEB88099E944E3?sequence=1.

See Lasker, supra note 18.

See, e.g., Meds for Missions, MAP International, https://www.map.org/medicines. Accessed Nov. 11, 2018.

See Bauer I, supra note 18; see also Bradke A, supra note 16.

Loiseau B, et al. ‘Don’t make my people beggars’: A developing world house of cards. Community Development J. 2016; 571(51): 571–584.

Kitsis E, Goldsammler M. Physician shadowing: A review of the literature and proposal for guidelines. Academic Medicine. 2013; 102(88): 102–110; Geiderman JM. Observers in the medical setting. Annals of Emergency Medicine. 2016; 86(70): 86–92.

The Future Docs Abroad Experience. Future Docs Abroad. https://allaccessmed.com/about/. Accessed Nov. 11, 2018.

The so-called “presumption against extraterritoriality” means that, absent clearly expressed congressional intent to the contrary, federal laws will be construed to have only domestic application. See Morrison v. National Australia Bank Ltd., U. S. 2010; 247(561): 255; see also Morrison and Kiobel v. Royal Dutch Petroleum Co., U. S. 2013; 108(569).

See Evans T, et al., supra note 9; Smaldino, supra note 21.

See Parry & Grant Encyclopaedic Dictionary of International Law (3d ed. 2009).

Gostin LO, et al., The Normative Authority of the World Health Organization. Public Health 2015; 854(129): 854–856.

Crump JA, et al. Global health training: Ethics and best practice guidelines for training experiences in global health. American J Tropical Medicine and Hygiene. 2010; 1178(83): 1178–1182.

Annotated Code of Maryland, Health Occupations, § 14–101(n) (West 2018).

“Position of the Federation of State Medical Boards In Support of Postgraduate Training and Licensure Standards,” Adopted as policy by the Federation of State Medical Boards in 1998 (on file with authors).

Whalen T, Wendel G. New supervision standards: Discussion and justification. Chap. 6 Monograph: The ACGME 2011 Duty Hour Standard Enhancing Quality of Care, Supervision. and Resident Professional Development. Available at: https://www.acgme.org/Portals/0/PDFs/jgme-11-00-39-45[1].pdf. Accessed Dec. 4, 2018.

See Int’l Ass’n Medical Regulatory Authorities, https://iamra.com/. Accessed Nov. 11, 2018.

Temporary Licence for Foreign Doctors, Kenya Medical Practitioners and Dentists Board. http://medicalboard.co.ke/temporary-licence-for-foreign-doctors/. Accessed Nov. 28, 2018. Other nations have similar temporary licensure laws. See Requirements for Registration and Licensure, Uganda Medical and Dental Practitioners Council, http://umdpc.com/forms.php. Accessed Nov. 11, 2018. The Liberia Medical & Dental Council allows visiting physicians to obtain a temporary license upon application and interview. The application for a temporary license is on file with the authors.

ASEAN Joint Coordinating Committee on Medical Practitioners. https://asean.org/wp-content/uploads/2012/05/AMS-Policy-for-Temporary-Licensing-and-Registration-Updated-AJCCM-22F.…pdf. Accessed Nov. 28, 2018.

Registration Process, Medical Council Malawi, http://www.medicalcouncilmw.org/mcm/process.php. Accessed Nov. 11, 2018.

Theoharis M. Practicing Medicine without a License, Criminal Defense Lawyer, https://www.criminaldefenselawyer.com/resources/practicing-medicine-without-a-license.htm. Accessed Nov. 28, 2018.

See The Medical and Dental Practitioners Act, Cap 221 Laws of the Federal Republic of Nigeria,1990 available at https://www.mdcn.gov.ng/page/about-us/mdcn-act-other-regulation; *see also* Rwanda Gazette Nº 44/2012 of 14/01/2013 (page 47), Law on the organisation, functioning and competence of the Medical and Dental Council. Available at http://www.moh.gov.rw/fileadmin/templates/HLaws/Medical_and_Dental_Council1.pdf (“Any medical doctor or dentist who practices his/her profession without being registered in the register of the Council … shall be brought before the competent courts of law.”).

See Bradke A, supra note 16.

Willgress L. Students volunteering abroad may be “burdens”, medic suggests as she sues organisation over Madagascan trip. The Telegraph. Accessed August 19, 2017, https://www.telegraph.co.uk/news/2016/08/19/students-volunteering-abroad-may-be-burdens-medic-suggests-as-sh/.

See Kitsis EA. Shining a light on shadowing. JAMA. 2011; 1029(305): 1029–1030.

Practice Standard. College Physicians and Surgeons British Columbia Accessed Sept. 1, 2017, https://www.cpsbc.ca/files/pdf/PSG-Job-Shadowing-Observing.pdf.

Kitsis EA, Goldsammler M. Physician shadowing: A review of the literature and proposal for guidelines. Academic Medicine. 2013; 102(88): 102–10; Geiderman JM. Observers in the medical setting. Annals Emergency Medicine. 2016; 86(70): 86–92.

See supra Crump, note 37.

DeCamp M, et al. Ethical obligations regarding short-term global health clinical experiences: An American College of Physicians position paper. Annals of Internal Medicine. 2018.

American Association of Medical Colleges. Guidelines for premedical and medical students providing patient care during clinical experiences abroad. 2011. Available at https://www.aamc.org/download/181690/data/guidelinesforstudentsprovidingpatientcare.pdf.

International Standards & Guidelines and Health Missions. Best Practices in Global Health Missions Accessed July 2017, https://www.bpghm.org/wp-content/uploads/2017/05/ISGsandHealthMissions.pdf. Accessed Nov. 29, 2018.

See, e.g., Roberts M. Duffle bag medicine. JAMA. 2006; 295(13): 1491–1492.

Ibid; see also Burris S, et al. Legal aspects of providing Naloxone to heroin users in the United States. Int J Drug Policy. 2001; 237(12); see also Davis C, Carr D. State legal innovations to encourage Naloxone dispensing. J Am Pharmacists Assn. 2017; S180(57): S180–84.

Ibid.

Meds for Missions. MAP Intern’l, https://www.map.org/medicines. Accessed Nov. 11, 2018.

State Prescription Drug Return, Reuse, and Recycling Laws. Nat’l Conference State Legislatures Oct. 1, 2018, http://www.ncsl.org/research/health/state-prescription-drug-return-reuse-and-recycling.aspx.

Ibid.

Ibid.

World Health Organization. Guidelines for Medicine Donations, World Health Org. 2011, http://apps.who.int/iris/bitstream/handle/10665/44647/9789241501989_eng.pdf;jsessionid=23F7DA3C2136B6D735AEB88099E944E3?sequence=1.

Ibid.

Ibid.

U.S. Food & Drug Administration. Questions and answers for the pub.: Donating drugs to international humanitarian relief efforts. U.S. Food & Drug Admin. https://www.fda.gov/downloads/NewsEvents/PublicHealthFocus/UCM249617.pdf. Accessed Nov. 19, 2017.

Ibid.

See 81 FR 97029, Dec. 30, 2016; see also U.S. Department of Justice. Emergency Assistance with a Domestic (or International) Disaster. U.S. Department of Justice. https://www.deadiversion.usdoj.gov/disaster_relief.htm. Accessed Nov. 11, 2018.

See Harrison L. Dentist arrested for trying to take drugs to Haiti. Dr.Bicuspid.com (May 3, 2010). https://www.drbicuspid.com/index.aspx?sec=ser&sub=def&pag=dis&ItemID=304512.

U.S. Customs and Border Protection. Traveling with medication. Nov. 14, 2016. https://help.cbp.gov/app/answers/detail/a_id/1160/kw/pharmaceutical.

See Guidelines for Medicine Donations, supra note 63.

Ibid.

Ibid.

Ibid.

See supra Evert, note 3.

Ibid.

Restatement (Third) of Torts: Liability for Physical Harm § 3 (P.F.D. No. 1, 2005); see Bradke A, supra note 16, at 31–36 (discussing standard of care for medical mission personnel).

See Elgafi S. Medical liability in humanitarian missions. J Humanitarian Assistance. 2014, https://sites.tufts.edu/jha/archives/2111.

Ibid.

Marcus D. Are you protected from liability when volunteering at home, abroad? AAP News June 28, 2010 (quoted in Uejima T, Medical Missions and Malpractice: The Current State of Medical Malpractice Overseas. 75 Am Soc Anesthesiologists. 2011; 22: 22–24).

The Center for Medical Missions study was reported in Uejima T. Medical missions and malpractice: The current state of medical malpractice overseas. Am Soc Anesthesiologists. 2011; 22(75): 22–24.

World Medical Association. WMA Statement on Medical Liability Reform. World Medical Ass’n April 2015, https://www.wma.net/policies-post/wma-statement-on-medical-liability-reform/.

See Elgafi S. Medical Liability in Humanitarian Missions, J. Humanitarian Assistance. Accessed 2014, https://sites.tufts.edu/jha/archives/2111.

Ibid.

Selcoe J. Answering the call to service: Encouraging volunteerism by protecting doctors as we protect ourselves. Texas Law Review. 2016; 94: 1265.

Uejima T. Medical missions and malpractice: The current state of medical malpractice overseas. Am Soc Anesthesiologists. 2011; 22(75): 22–24.

Ibid.

Evert J, et al. Do you GASP? How pre-health students delivering babies in Africa is quickly becoming consequentially unacceptable. Health Advisor. 2015; 61(35): 61–65.

Document on file with authors.

See https://sua.umn.edu/groups/management/registration/classification-chart/.

See https://www.healthcareers.umn.edu/courses-and-events/online-workshops/global-ambassadors-patient-safety.

See Carlson D, Thompson JN. The role of state medical boards. AMA J Ethics. 2005; 311(7): 311–14.

FDA report. Questions and Answers for the Public Donating Drugs to International Humanitarian Relief Efforts, https://www.fda.gov/downloads/newsevents/publichealthfocus/ucm249617.pdf.

QUIP, Forum on Educ. Abroad, https://forumea.org/get-involved/quality-assurance-programs/quip/. Accessed Nov. 11, 2018.

Accreditation and Standards. Am. Camp Ass’n, https://www.acacamps.org/staff-professionals/accreditation-standards. Accessed Nov. 11, 2018.

We Received the Gold Seal of Approval from the Joint Commission, Joint Commission, https://www.jointcommission.org/assets/1/18/TJC_GoldSeal_brochure.pdf. Accessed Nov. 11, 2018.

Green T, et al. Perceptions of short-term medical volunteer work: A qualitative study in Guatemala. Globalization and Health. 2009; 1(5): 1–13, https://globalizationandhealth.biomedcentral.com/track/pdf/10.1186/1744-8603-5-4; Kraeker C, Chandler C. “We learn from them, they learn from us”: Global health experiences and host perceptions of visiting health care professionals. Academic Medicine. 2013; 483(88): 483–487.

